# A Great Enterprise

**Published:** 1871-10

**Authors:** 


### A GREAT ENTERPRISE.

Going through the streets of Montreal, recently, an evening paper was noticed in the hands of many persons in the public square, at the hotels, and by the wayside. Citizens read it, porters read it, coachmen read it, loafers read it. On asking a boy for one, and the price, — "Five cents."

"You little monkey, do you ask five cents for a small evening newspaper in Canada? What is that?" pointing to the printed price of "one cent." Seeing that although a "foreigner" we knew the price, he was glad to take a cent for an evening paper about as large as the "New York Sun." It was the "Montreal Daily Witness," a religious daily newspaper; and then our mind ran back to a small evening paper which had just begun to make its appearance in New York, about the time of our leaving, called the "New York Witness." Price one cent. A religious daily evening newspaper for one cent. The Montreal paper has proved a pecuniary success, because it has been conducted with ability, judgment, and enterprise; the proprietor is now attempting to establish a similar paper in New York city. The first edition is issued at twelve o'clock every day, and two or three subsequent editions during the afternoon; it contains the latest and most important telegrams, a succinct collection of all the main items of news in the city and out of it, and in addition, a judicious gathering of short moral, religious, scientific, and practical articles on all subjects. No boy or girl, woman or man can read a copy without being instructed, benefited, elevated. Some of the headings of the issue for this day, Sept. 2d, are: Golden Hills, a story; Bible; Marriage Tie; Religious Poetry; Future State; Haste and Health; Prohibitory Law; Emily Faithful; The Young Woman's Friend; Relief in Trouble; French Wine Drinking; Longevity; besides a large amount of city and country and foreign and domestic news. No nation can be saved from a gradual degradation, unless the masses have reading which elevates, instructs, and purifies; and while so many of the daily papers and weekly issues, of pictorials and the like, abound in all sorts of mental and moral nastiness, not only in the reading matter, but in the advertisements, it is certainly high time that some antagonistic influences should be set in operation. The proprietor of the "New York Daily Witness" has set about this single handed and alone; and if all the good people of New York and its vicinity do not encourage him by taking the paper and giving him a liberal share of their advertising patronage, it will be a blushing shame and an indelible disgrace.

There are several ways in which the success of this admirable paper can be forwarded.

Call out for the "Daily Witness" whenever you want a paper; it is only a cent, and then buy some other if you desire it. By this inquiry you draw the attention of others to it; and in proportion as you are respectable in appearance, manner, and deportment, you add influence to the paper itself.

Advertise in it, at one cent a line, and thus give the influence of your name in the most public manner, to the promotion of an enterprise which is worthy of encouragement from all substantial citizens.

Let all our churches which advertise their meetings, do it in the "Witness," and then the poor have it in their power to obtain the information at the smallest cost on Saturdays, and have a good deal of solid Sunday reading besides.

Let our Christian bankers and merchants advertise in the "Witness" and take it too, even if it be for the servants to read in the kitchen, after it has been read in the parlor, and the names of these men will very naturally draw the attention of others to the "Witness" as an advertising medium.

The editor and proprietor of the "New York Daily Witness' is a man of high social position, of cultivation, and financial ability; he comes to us with high recommendations from some of the most distinguished commercial, literary, and professional men in the New Dominion, and we earnestly trust that he will meet with a warm welcome and a liberal patronage, so that a circulation which has risen to over eleven thousand in a few weeks may, in a few months, reach ten times eleven thousand, to interest, amuse, and instruct as many families by its daily visits. There are tens of thousands of farmers who, if they were to take it and have it read every night as the family are gathered around the centre-table or the winter fireside, would find themselves more and more interested in what is going on in the world; the number of subjects for reflection, for conversation and remark, would be increased, and soon that insanity-producing monotony of farm life would be broken up, and a general elevation and ambition and enterprise would be in time an inevitable result.

We cannot say that in a moral and religious point of view the daily press of large cities is good. Pio Nono is not altogether wrong in wishing that the daily newspapers in Rome should not find their way into Roman Catholic families. Blasphemous, scandalous, incendiary, and obscene newspapers ought not be allowed in families; that there are such daily cheap papers in our large cities is beyond contradiction. It is only when the reading matter of a newspaper is on the side of decency and good morals, and law and order, always, that it is a family blessing. There is a vast amount of clap-trap and buncombe in the advocacy of free speech, free press, and universal education, but there are metes and bounds to these things, and when the press and the speech become licentious and profane, then it becomes a curse. And it becomes a practical question to be instantly decided upon by every man to whom it is proposed, Am I not bound to patronize a daily paper which is always on the side of good morals, of religion, and of the Sabbath day?

